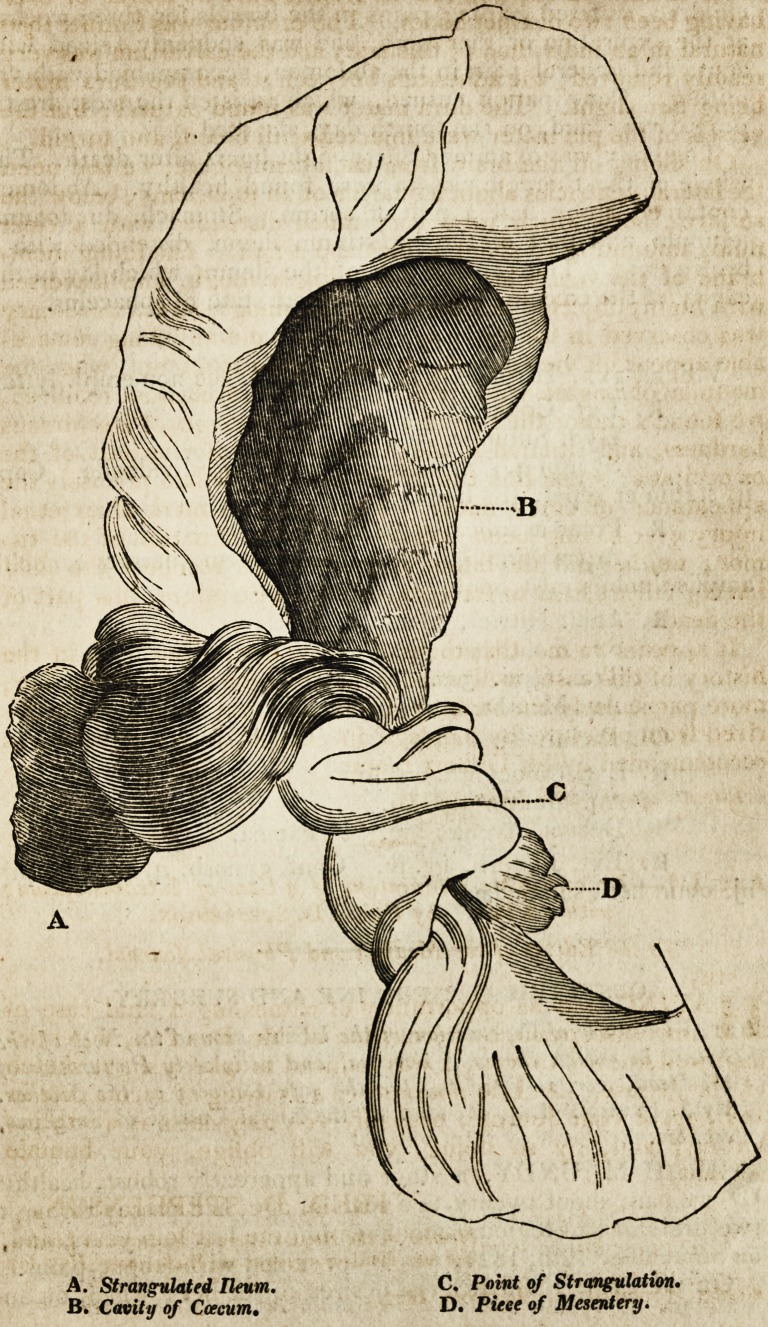# Necrographic Delineation of a Case of Introsusception

**Published:** 1822-04

**Authors:** Fred. D. Stephenson

**Affiliations:** Member of the Royal College of Surgeons in London, and late House-Surgeon to the Westminster Hospital. Alcester, Warwickshire


### Art. III.—*Necrographic Delineation of a Case of Introsusception; with a Plate.* By Fred. D. Stephenson.

*To the Editor of the Medical and Physical Journal.*

SIR,

HAVING had an opportunity of witnessing a fatal case of introsusception, by permission of Mr. Purton, a gentleman well known to you, I was induced to take a slight sketch of the disease. As I do not observe a drawing of such a case in any part of your Journal, probably you may deem it worth inserting; and, by so doing, you will oblige, your humble servant,

FRED. D. STEPHENSON,

*Member of the Royal College of Surgeons in London, and late House-Surgeon to the Westminster Hospital.*

*Alcester, Warwickshire; March 6th, 1822.*

**A.** *Strangulated Ileum.*

**B.** *Cavity of Cœcum.*

**C.** *Point of Strangulation.*

**D.** *Piece of Mesentery.*

Mary Canning, æt. 50, of a weakly habit, the mother of eight children, suffered griping pains in the bowels for three weeks: at the expiration of that time, she was suddenly seized with acute and general pain in the abdomen, accompanied with vomiting. Constipation ensued, which resisted the most drastic purgatives; and, on the fourth day, she died.

The body was examined thirty-eight hours after death. The whole of the thoracic viscera were found healthy. Abdomen contained about half a pint of serum. Stomach, duodenum, jejunum, and part of the intestinum ileum, distended with a brown fluid. The greater part of the ileum, which lay in the cavity of the cœcum, was in a complete state of sphacelus.

TREATMENT.

V.S. ad ℥xx. Hirud. xij. hegion. umbil. nec non empl. lyttæ.

℞. Ext. Colocynth. co. ʒj.

Hyd. Submur. ʒss.

Syrupi q. s. ut ft. massa in pil. xij. dividenda. Cap. ij. statim et repet. 4tis horis.

℞. Potassæ subcarb. ʒiss.
Aquæ puræ, ℥ij. M. sumat partem quartam c. cochl. amplis duobus mist. sequent. pro re nata.

℞. Acid. Nitrici, ʒjss.

Sp. Æther. co. ʒjss.

Træ. Opii. ʒss.

Aq. Menthæ sativ. ℥jss. M.

Ol. Ricini, ℥j. statim.

℞. Ext. Colocynth. co. ʒj.

Aq. Menth. pip. ℥ij.

Decoct. Avenæ, ℥vj. F. enema.

℞. Ext. Elaterii, gr. iv. Conf. cynosb. q. s. ut ft. pil. iij. omni horâ sumendæ.

## Figures and Tables

**Figure f1:**