# Errata in Mr. Stephenson's Paper, in Last Number

**Published:** 1822-05

**Authors:** 


Errata *in* Mr. Stephenson's *Paper, in last Number.*

Line 14, p. 276,—region.

— 22, — —acid. citrici.

— 31, — —℞. Ext. Elaterii, gr. x. Conf. q. s. ut ft. pil. iij. Capt. j. omni horâ.

